# Supplementary material for: Light-tuned selective photosynthesis of azo- and azoxy-aromatics using graphitic C3N4
Source: Nat Commun. 2018 Jan 4;9:60. doi: 10.1038/s41467-017-02527-8 (PMC5754351; doi:10.1038/s41467-017-02527-8)
Supplement: Supplementary file 1 — Supplementary Information [file 41467_2017_2527_MOESM1_ESM.pdf]

Supplementary Methods

The g-C_3N_4 was synthesised by a typical pyrolysis method with urea as the precursor[1]. Urea (analytic grade, Shanghai Aladdin Bio-Chem Technology Co., Ltd.) was used as received without further purification. Urea powder was loaded into a crucible with a lid and calcined in a muffle oven at 550 ^{o}C for 2 h using a ramping rate of 1 $^{o}C\cdot min^{-1}$ and then cooled to room temperature with a rate of 5 $^{o}C\cdot min^{-1}$. For the scaled-up experiment a total amount of 0.5 kg of the g-C_3N_4 photocatalyst was synthesised using two 100 mL crucibles (80 g urea in each, purchased from Sinopharm Chemical Reagent Co., Ltd.) and by repeating the process for 60 times.

The A-g-C_3N_4 sample was prepared according to a literature procedure[2] by post heating of the as-prepared g-C_3N_4 at 630 ^{o}C for 2 h. A ramping rate of 2 $^{o}C\cdot min^{-1}$ was used under Ar atmosphere (Ar flow: 50 $mL\cdot min^{-1}$) followed by natural cooling to room temperature. As shown in Supplementary Figure 1 the yellow g-C_3N_4 was converted to orange A-g-C_3N_4 after the heat treatment.

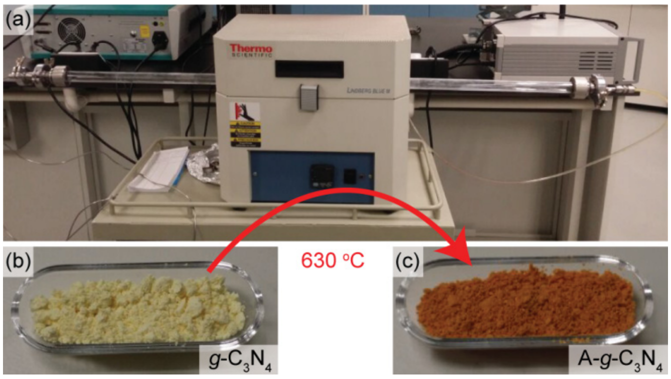

Supplementary Figure 1 Photocatalyst preparation. Photographs of the as-prepared g-C_3N_4 and A-g-C_3N_4 that is derived from g-C_3N_4 *via* annealing at 630 ^{o}C for 2 h under a flow of Ar at ambient pressure.

The photocatalytic nitrobenzene reduction reactions were performed in leak tight reactors connected to a stainless steel vacuum/gas line, as shown in Supplementary Figure 2. The reaction chamber is composed of (A) a glass reactor with a stainless steel adapter (KF-40 with two 1/16" Swagelok® adapters, 10 mL of reactants/catalysts suspension) and (B) a quartz viewport. The reactor is connected to the vacuum line (C) through a gas valve (D). The LED lamp (E) is placed on top of (B). The vacuum/gas line is connected to a rotary pump and a N_2 gas bottle (purity = 99.999%) through the bellow and the inlet gas line (F), respectively. During the reaction, (A) was immersed into a water bath to keep the temperature constant (RT).

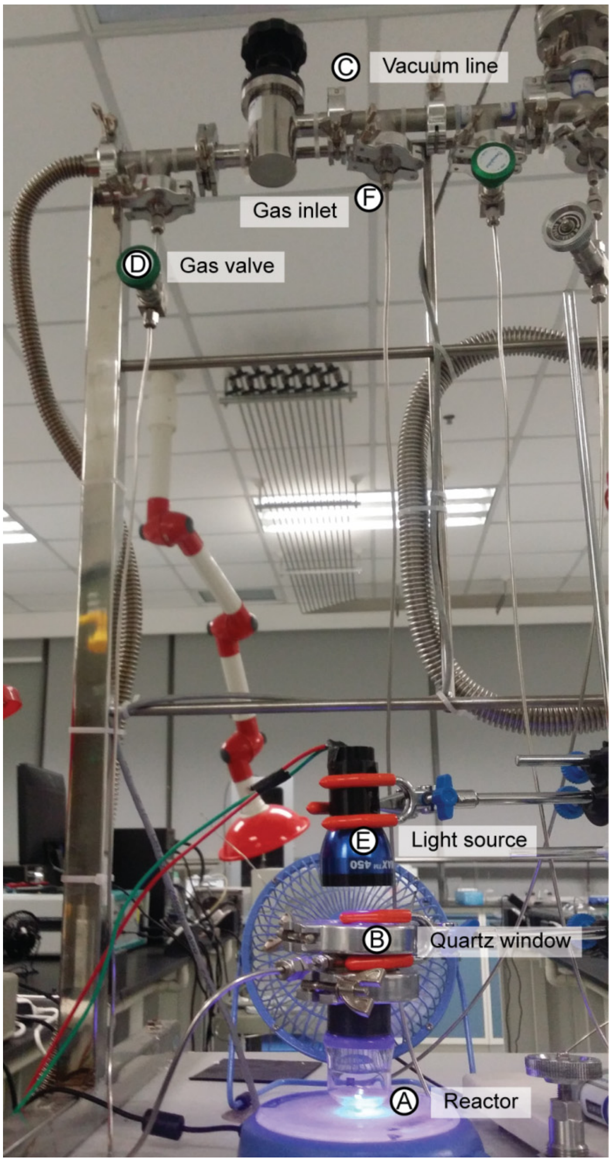

Supplementary Figure 2 . Image of our home-built vacuum-gas line and a reactor for photocatalytic tests.

A reaction mixture is consisted of 50 mg photocatalyst, 80 μmol of a nitroarene, and 100 μmol KOH in 10 mL isopropanol. All chemicals were of analytic grade and used as received without further purification (Shanghai Aladdin Bio-Chem Technology Co., Ltd.). The reaction chamber was vented and N_2 purged for 3 times, before the suspension was irradiated by a monochromatic LED lamp (365 nm, 410 nm, or 450 nm Spectroline®) under a 1 bar N_2 atmosphere. All lamps were characterised with a full-width-at-half-maximum (FWHM) of 10 nm according to the manufacturer. The liquid phase products were analysed by gas chromatography (Agilent GC 7890B) and combined gas chromatography and mass spectrometry (GC-MS, Agilent 6890N network GC system coupling with 5973 Network Mass selective Detector). The GC system was equipped with an HP-5MS column, 30 m × 0.250 mm × 0.25 μm and an FID detector. The GC and GC-MS analyses were performed to determine the conversion and selectivity of the photocatalytic reduction reaction. The nitroarene conversion is defined as the molar percentage of the consumed reactant, while the selectivity of the desired product is determined as the molar percentage of the total products. Moreover, the yield corresponding to the azo- or azoxy-aromatic product refers to the GC yield, which is based on the above nitroarene conversion and the desired product selectivity.

In the following, quantum efficiency (QE) calculations are described for the selective photocatalytic reduction of nitro-aromatic precursors. From Supplementary Equation (1), the number of incident photons per hour (N_{1h}) was determined.

$$N_{1h} = \frac{E*\lambda*t}{h*c} = \frac{W_{lamp}*S_R*\lambda*3600}{6.626*10^{-34}*3*10^{8}} \qquad (1)$$

where W_{lamp} is the light intensity of the applied LED lamps measured by an optical power meter (0.03 W·cm^{-2}), S_R is the effective irradiation areas (12.56 cm^2 for the lab-scale reactions) and λ is the light wavelength of the irradiation light (in nm). The estimated N for 410 nm (N_{410}) and 450 nm (N_{450}) irradiation were 2.8x10^{21} h^{-1} and 3.1x10^{21} h^{-1}, respectively.

The QE can be calculated according to Supplementary Equation (2), in which $n_{e,i}$ is the number of electrons needed for one desired product molecules.

$$\mathrm{QE} = \frac{\Sigma_i n_{e,i} * n_p}{N_{1h} * t_R} * 100\% \qquad (2)$$

where $n_{e,i}$ = 6, 8, and 6 for the production of one aromatic azoxy-, azo-, and amine molecule; n_p is the number of molecules of the corresponding products; t_R is the irradiation time (h).

Supplementary Table 1. The effect of irradiation wavelength on the photo-reduction of nitrobenzene.

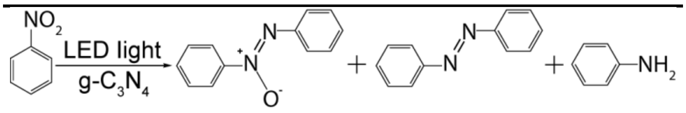

(I) (II) (III)

Light Wavelength / nm	Con. / %	Sel. / %			Yield / %		
		I	II	III	I	II	III
410	95	6	94	<1	6	89	<1
450	97	95	5	<1	92	5	<1
365	82	33	57	5	27	47	4

Reaction conditions: 50 mg g-C_3N_4, 80 μmol nitrobenzene and 10 ml 0.01 mol/L KOH isopropanol solution under a 1 atm N_2 atmosphere and irradiated by LED lamps with different wavelengths: 365 nm (0.02 W·cm^{-2}) for 8 h, 410 nm (0.03 W·cm^{-2}) for 5 h and 450 nm (0.03 W·cm^{-2}) for 12 h. I: azoxybenzene, II: azobenzene, III: aniline.

The effect of the irradiation wavelength on the conversion and selectivity of the photocatalytic nitrobenzene reduction was investigated by using monochromatic LED lamps with emission peaks at 365 nm, 410 nm and 450 nm, respectively, as shown in Supplementary Table 1. Whilst a high conversion (≥90%) was achieved by applying light with different irradiation wavelengths, the selectivity varied significantly. The use of blue light (450 nm, 12 h) led to successful synthesis of azoxybenzene (selectivity of 90%), the use of purple light (410 nm, 5 h) resulted in azobenzene with a high selectivity (94%). In comparison, the main products were still azo- and azoxy-benzene (sel. to [azo + azoxy] ≥ 90%) at a high conversion of nitrobenzene (~ 80%) under UV irradiation (8 h, Supplementary Table 1). Therefore, it is evident that the g-C_3N_4 photocatalyst system promotes the N-N coupling of nitrobenzene and suppressed the full reduction to aniline under all irradiation conditions.

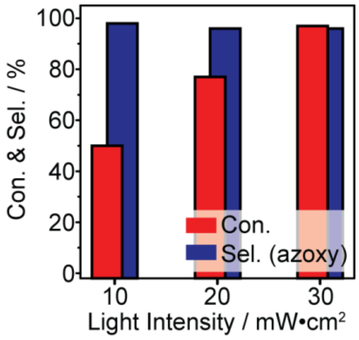

Supplementary Figure 3 The effect of the light intensity on the photo-reduction of nitrobenzene. Reaction conditions: 50 mg g-C_3N_4, 80 μmol nitrobenzene and 10 ml 0.01 mol·L^{-1} KOH isopropanol solution under a 1 atm N_2 atmosphere and irradiated by a 450 nm LED lamp with different intensity for 12 h (10, 20, 30 mW·cm^{-2}).

The effect of light intensity was studied using a 450 nm LED lamp with tuneable output power, which was controlled by changing the input voltage and current (Supplementary Figure 3). While the conversion of nitrobenzene (after 12 h irradiation) could be enhanced from 50% to 75% and eventually to 97% as the light intensity was gradually increased from 10 to 20 and further to 30 mW·cm^{-2}, while the high selectivity to azoxybenzene (≥ 95%) was maintained.

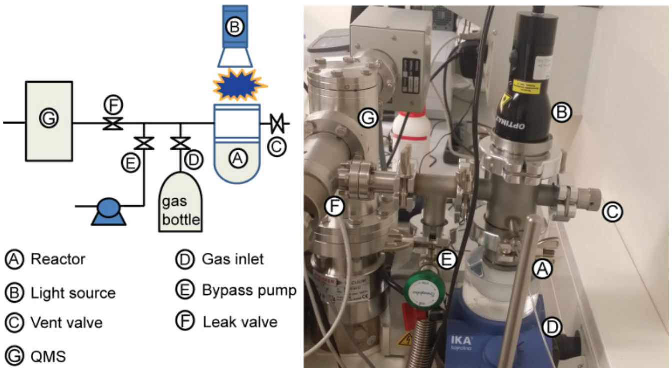

Supplementary Figure 4. Scheme and image of the *in-situ* mass spectrometer based reactor[3,4].

It was investigated if any gas phase product(s) evolved during the photo-reduction of nitrobenzene. The gas phase was analysed quantitatively using a home-built *in-situ* mass spectrometer (MS) based reaction system (Supplementary Figure 4)[3, 4]. The main setup can be divided into three parts: A reaction chamber that can be evacuated with a bypass pump or pressurised by a gas inlet valve; a leak valve that is normally regulated to a leaking rate of $\sim 1\times10^{-5}$ mbar·L·s^{-1} (total gas consuming rate of 1.6 μmol·h^{-1}) for quasi steady state sampling of the partial pressure of any gas in the reaction chamber; and a quadruple mass spectrometer (HPR-20, Hiden) for precise detection of the partial pressures of target gases. Since the system has a well-defined volume and is leak tight, the quantity of evolved gases can be calculated based on their partial pressure by applying the ideal gas law with proper calibration of the MS sensitivity factor. Our *in-situ* MS based reaction system can be employed to monitor the evolution of several gases (*i.e.*, H_2, O_2, CO_2, CO and CH_4) all at once.

Supplementary Figure 5 shows the time-resolved mass signals during the photocatalytic reduction of nitrobenzene by g-C_3N_4 under continuous 410 nm light irradiation for 5 h (marked as region IV), as the partial pressures of the gases remain constant. It is clear that no oxygen (m/z = 32) or hydrogen (m/z = 2) were produced during the full duration of the light-driven reaction (region IV). This suggests that excess oxygen from nitrobenzene was removed by the adsorbed H (H_{ads}) on the g-C_3N_4 surface in the form of water or OH^- rather than O_2 as previously reported[5].

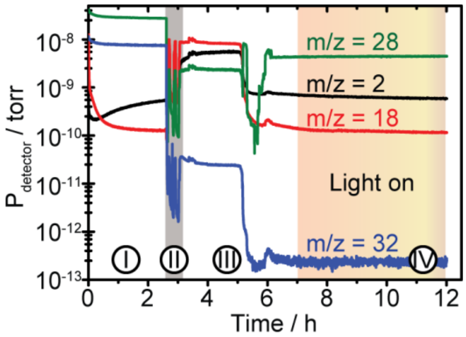

Supplementary Figure 5 Time-resolved mass spectra during the photocatalytic nitrobenzene reduction. The four regions represent (I) Stabilisation of the MS, (II) Degassing and N_2 purging, (III) System stabilisation (caused by water vapour), and (IV) Irradiation/photosynthesis. Masses of 18, 28, 32 and 2 can be assigned to water, nitrogen, oxygen and hydrogen, respectively. Reaction conditions: 50 mg g-C_3N_4, 80 μmol nitrobenzene and 10 mL 0.01 mol·L^{-1} KOH isopropanol solution under 1 atm N_2 atmosphere. Irradiation: 410 nm LED lamp, 30 mW·cm^{-2}, 5 h.

The depletion/reduction of dissolved oxygen by the photocatalyst without the presence of nitrobenzene (Manuscript Fig. 2a) was performed using the *in-situ* MS system. The reaction was performed under vacuum conditions ($\sim 10^{-3}$ mbar) with an initial O_2 partial pressure adjusted to $2\text{x}10^{-11}$ mbar (determined by the MS, corresponding to 28 ppm of residual O_2) prior to light irradiation/reaction. In short, we employed evacuation (instead of purging by N_2) to control the initial O_2 concentration; all other reaction parameters were identical to that of the nitrobenzene photoreduction process.

The prices of different catalysts shown in Table 1 of the manuscript were estimated according to the catalyst compositions presented in the cited studies (Supplementary Table 2). The preparation of g-C_3N_4 was realised by a simple one-pot method that only involves the precursor (urea), a crucible, and a muffle furnace operated in air. All the other photocatalysts listed in Table 2 were synthesised under more complicated and/or harsh conditions with the use of several organic modifiers, a more advanced procedure, several preparation steps, and/or the need of a flammable gas (*e.g.* H_2). In order to simplify the estimation of the catalyst price, we only considered the prices of the commercially available precursor chemicals and ignored the processing costs.

Supplementary Table 2. Price estimation of the photocatalysts listed in Table 1.

Photocat.	Component[a]	SKU-Packing[b]	Unit Price (€·g-1)[c]	Price (€·g-1 cat.)[d]	Total Price (€·g-1)[e]
SiO_2/ CdS/Rh	SiO_2	-	-	-	7.7
	CdS	217921-20G	6.2	6.1	
	$RhCl_3 \cdot xH_2O$	206261-1G	263.0	1.6	
Au/ TiO_2	Chloroauric acid	520918-1G	151.5	13.0	14.4
	TiO_2	718467-100G	1.5	1.4	
Cu/ Graphene	Copper acetate	229601-10G	20.1	3.2	92.2
	Graphene oxide	GF15433253-1EA	93.7	89.0	
Au/ TiO_2-Ag	Chloroauric acid	520918-1G	151.5	6.1	7.6
	TiO_2	718467-100G	1.5	1.4	
	Ag_2SO_4	497266-10G	9.4	0.1	
TiO_2/ N_3/ K_2PtCl_6	TiO_2	718467-100G	1.5	1.2	57.1
	N_3 Dye	703206-1G	372.5	55.9	
	K_2PtCl_6	-	-	-	
Ni_2P/ CdS	Ni_2P	372641-10G	8.4	0.4	6.2
	CdS	217921-20G	6.2	5.8	
g-C_3N_4	Urea	10023292-500G[f]	0.004	0.1	0.1

a: Commercial precursors involved in the photocatalyst syntheses. *b,c*: The stock keeping unit (SKU)-packing size and unit price (Mar. 2017) can be found on the website of Sigma-Aldrich Co. LLC. *d*: Calculated according to the contents of each constituent in one gram of the final catalysts. *e*: Determined from the summation of the prices for each involved constituent. *f*: From Sinopharm Chemical Reagent Co., Ltd.

The 800 mL scaled-up experiment was carried out using a 1 L three-neck round-bottom glass flask (borosilicate 3.3, >90% transmission of visible light, the diameter of the flask is d_{flask} = 11 cm), as shown in Supplementary Figure 6a. The total irradiated surface area was estimated to be 255 cm^{-2} by considering half of the side area when filled with 800 mL of liquid (8.5 cm in height, 160 cm^2) and the upper surface of the liquid (95 cm^{-2}). The three necks were used for N_2 bubbling, a gas outlet, and a thermocouple, respectively. The reaction suspension consisted of 4 g of g-C_3N_4 and 8 mM of nitrobenzene in an 800 mL solution of 0.01 $mol \cdot L^{-1}$ KOH in isopropanol. The reaction was performed on the roof of the SynCat@Beijing building in the Huairou district, Beijing, for 3 days (starting on the 30th of September 2016 and ending on the 2nd of October 2016) under solar irradiation (Supplementary Figure 6b). The sunrise and sunset time were 7 am and 5 pm on these three days, respectively. Since no nitrobenzene conversion was observed during the night, only daytime hours was counted as actual reaction time.

A 2 mL sample was withdrawn every 5 h, centrifuged, and analysed by GC. The reaction temperature varied from 18 to 28 ^oC during the whole process due to the solar irradiance /day time temperature change. The colour of the suspension gradually changed from yellow to orange during the experiment, indicating the formation of azoxybenzene. The phase pure azoxybenzene was obtained by a simple filtration, distillation and crystallisation process, as shown in Supplementary Figure 6c to 6e.

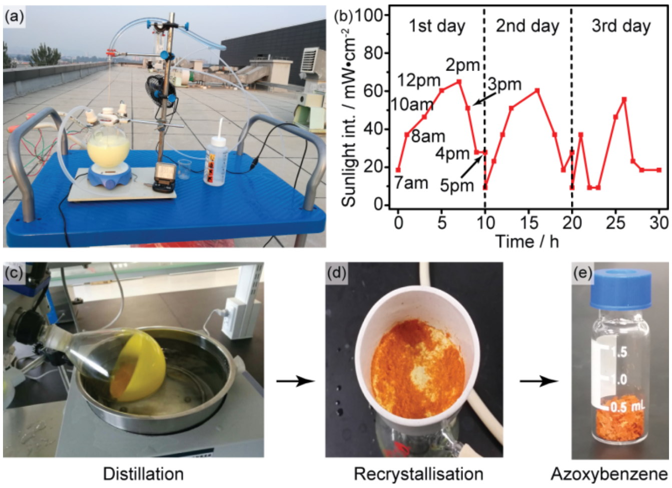

Supplementary Figure 6 The 800 mL scaled-up test. (a) Photograph of the 800 mL scaled-up reaction. (b) Time-dependent variation of the sunlight intensity. (c) Photograph of the separation process and the final crystallised azoxybenzene.

The time-dependent sunlight intensity was recorded during day time using an optical power meter (Newport 1918-R) to estimate the quantum efficiency (Fig. S6b). After integration of the sunlight intensity over the whole reaction time (30 h), the averaged input solar power was determined to be 38 mW·cm^{-2}. Due to the absorption properties of the g-C_3N_4 catalyst, only the fractions of sunlight with wavelengths smaller than 480 nm were considered in the determination of the QE as follows. According to the literature[6], the part of the solar spectrum in the UV to blue light range (from 280 to 480 nm) can be divided into three intervals: i = 1 is the zone from 280 to 400 nm with 340 nm as the average wavelength ($\lambda_{i=1}$); i = 2 covers the range from 400 to 440 nm with $\lambda_{i=2}$ of 420 nm; and i = 3 is located from 440 to 480 nm with 460 nm as $\lambda_{i=3}$. The following Supplementary Equation (3) was used to calculate the effective total incident photon flux per hour from the sun (N_{1h}^{sun}).

$$N_{1h}^{sun} = \sum_i \frac{E_{sun}*p_i*S_R*\lambda_i*t}{h*c} = \sum_i \frac{0.038*p_i*255*\lambda_i*3600}{6.626*10^{-34}*3*10^{8}} \quad (3)$$

where E_{sun} is the averaged sunlight intensity (0.038 W·cm^{-2}), p_i is the fraction of solar power in the intervals i = 1-3 (p_1=7.63%, p_2=5.08%, p_3=5.85%)[6], λ_i is the averaged wavelength of interval i and S_R is the effective irradiation area (255 cm^2).

The estimated N_{1h}^{sun} is 1.3*10^{22} h^{-1}.

The quantum efficiency can be therefore estimated by Supplementary Equation (4):

$$\text{QE} = \frac{\sum_i Con.*n_{nitro}*Sel._i*n_{e,i}}{N_{1h}^{sun}*t_R} * 100\% \quad (4)$$

where *Con.* is the conversion of nitrobenzene after 30 h (60%), $Sel._i$ is the selectivity of the corresponding product (90% for azoxybenzene, 5% for azobenzene, and 5% for aniline). n_{nitro} is the total number of nitrobenzene reactant molecules (0.008 M * 0.8 L * 6.02*10^{23}), $n_{e,i}$ is the number of electrons needed for converting one nitrobenzene to the corresponding product (3 for 0.5*azoxybenzene, 4 for 0.5*azobenzene, and 6 for 1*aniline).

The estimated QE is 1.9%.

We have further scaled up the reaction to 80 L using a single-walled 100 L glass reactor (borosilicate 3.3, Zhengzhou Greatwall Scientific Industrial and Trade Co. Ltd), as shown in Supplementary Figure 7a. The reactor is equipped with an electric motor (A) for stirring, a control box (B) for the motor and the thermocouple, and a gas bubbling tube (C) for N_2 purging. The reaction suspension consisted of 400 g g-C_3N_4 and 8 mM of nitrobenzene in 80 L of 0.01 mol·L^{-1} KOH isopropanol solution. The reaction was carried out on the roof of a building in Shenzhen for 23 days (from 18th December 2016 to 9th January 2017) under solar irradiation. A 10 mL sample was withdrawn daily, centrifuged, and analysed by GC. We recorded solar intensity data several times every day by the use of a power meter. Subsequently, the daily sunlight power was integrated over time in the same manner as described above for the 800 mL reaction test to obtain the averaged time-dependent variation of sunlight intensity, Supplementary Figure 7b. Moreover, a typical solar emission spectrum was also recorded by the Newport 1918-R optical power meter on site as shown in Supplementary Figure 7c.

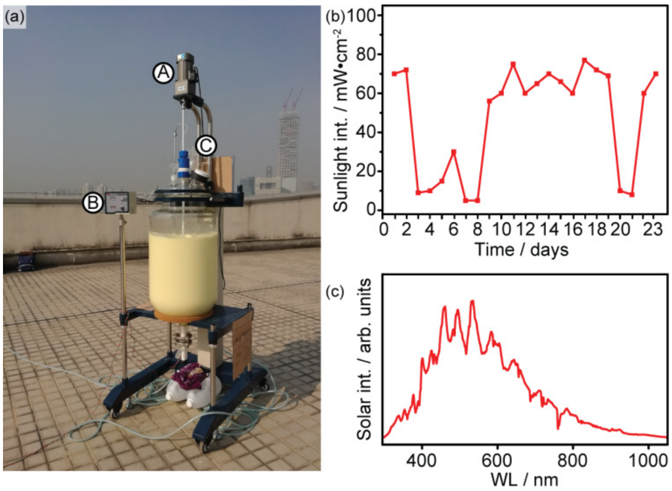

Supplementary Figure 7 The 80 L scaled-up test. (a) Photograph of the 80 L setup. (b) Time-dependent variation of the sunlight intensity. (c) The solar spectrum recorded during reaction.

Estimation of the scaling-up effect (800 mL to 80 L): During the 3rd to 8th day and on the 20th to 21st day, the solar irradiation intensity was very low due to cloudy or rainy weather (Supplementary Figure 7b), and the reaction was almost stopped on those days (Fig. 1g). Therefore only the sunny days were counted as effective reaction time (~ 135 hours, that is 15 days with 9 hours per day). The averaged solar energy input was ~ 199 W as the effective irradiation area was 2967 cm^2 and the averaged unit solar power was 67 $mW \cdot cm^{-2}$. In contrast, the reaction time for the 800 mL scaled-up test was 30 hours and the averaged solar energy input was about 10 W (255 cm^2 of effective irradiation area and 38 $mW \cdot cm^{-2}$ of unit solar power).

The reaction time of the 80 L scale-up test is about 4.5 times longer than that of the 800 mL scaled-up test when they had both reached a conversion of ~60%. By considering differences in the solar energy input (199 W for the 80 L scaled-up test and 10 W for the 800 mL scaled-up test) and the quantity of nitrobenzene (640 mmol for the 80 L test and 6.4 mmol for the 800 mL test), we estimate that the reaction rates for the 80 L and 800 mL scaled-up tests are comparable.

The powder X-ray diffraction (PXRD) data were collected on all samples using a Bruker D8 Advance diffractometer equipped with a Cu-Kα source (40 kV, 40 mA). The PXRD patterns were recorded in θ-2θ geometry with a scan range from 5 to 60° with a step size of 0.04 ° and a scan rate of 0.1 $^{\circ} \cdot s^{-1}$. Supplementary Figure 8a shows the PXRD patterns of the as-prepared g-C_3N_4 and A-g-C_3N_4. For g-C_3N_4, the (100) and (002) diffraction peaks are observed at 2θ angles of 12.6° and 27.5°, respectively. The relatively sharp (002) diffraction peak suggests long-ranged stacking of the triazene layers into a graphite-like structure, whereas the weak (100) peak is indicative of some degree of periodicity within the layers[2, 7]. In contrast, the A-g-C_3N_4 is characterised by a much broader and weaker (002) peak and the absence of the (100) peak revealing that the material is disordered in agreement with the literature[2].

The Brunauer–Emmett–Teller (BET) surface areas of the as-prepared samples (g-C_3N_4 and A-g-C_3N_4) were determined by nitrogen adsorption/desorption isotherms measured at 77 K using a Micromeritics ASAP 2420 system, as shown in Supplementary Figure 8b. The g-C_3N_4 has a BET surface area of 49 $m^2 \cdot g^{-1}$ while the A-g-C_3N_4 attains a larger surface area of 112 $m^2 \cdot g^{-1}$, which could be due to the more porous nanostructure caused by the partial decomposition of the graphite-like stacking of g-C_3N_4 during the post-heating process.

The diffuse reflectance spectroscopy (DRS) shown in Supplementary Figure 8c were recorded at room temperature using a Hitachi spectrometer (UH4150). $BaSO_4$ (spectroscopy grade) was used as the reference and the measurements were performed in the wavelength range of 300-800 nm. According to the Kubelka-Munk theory[8], Tauc plots of an indirect semiconductor can be drawn by plotting $[F(R) \cdot hv]^{1/2}$ versus hv (incident photo energy)[9], where $F(R) = (1-R)^2/(2R)$ and R is the measured reflectance. As shown in Supplementary Figure 8d the bandgaps of our as-prepared samples can be estimated to the intersection point between the tangent to the inflection point and the vertical base line. The A-g-C_3N_4 sample shows an absorption tail that extends up to 600 nm, which corresponds to a narrow bandgap of 2.05 eV. In comparison, the g-C_3N_4 shows a much wider bandgap of 2.77 eV.

In addition, we measured the DRS of a 1 wt% Au supported on TiO_2 to compare the light absorption of TiO_2 and Au nanoparticles (NPs), as shown in the Supplementary Figure 9. The plasmonic absorption of the Au NPs (centred at ~550 nm) was much weaker than that of the TiO_2.

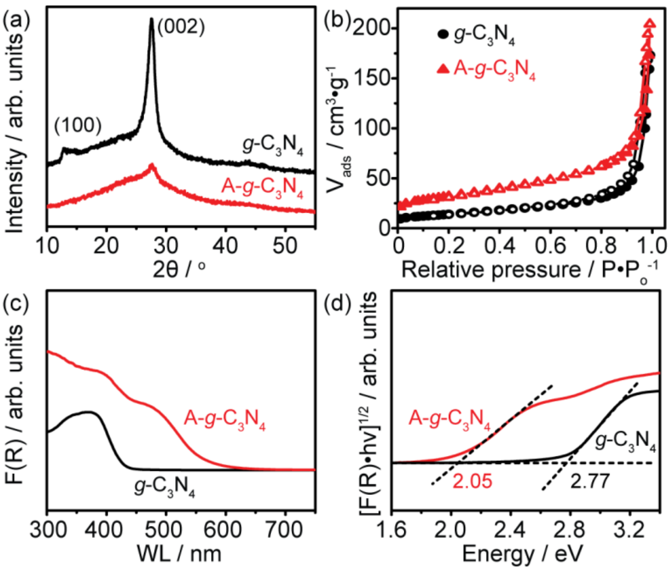

Supplementary Figure 8 Physical property analysis. (a) PXRD patterns of the as-prepared g-C_3N_4 and A-g-C_3N_4. (b) N_2 adsorption/desorption isotherms for g-C_3N_4 and A-g-C_3N_4. (c) and (d) DRS and Tauc plots of the as-prepared g-C_3N_4 and A-g-C_3N_4.

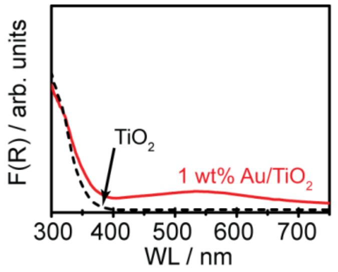

Supplementary Figure 9 Optical property analysis of TiO_2 based materials. DRS plots of the as-prepared 1wt% Au supported on TiO_2. The Au NPs were deposited on TiO_2 by a photo-deposition method.

The surface chemical composition of the samples were analysed using an X-ray photoelectron spectrometer (XPS, K-alpha, Thermo Fisher Scientific, USA). The instrument was equipped with a monochromatic Al Kα X-ray source. The survey scans were performed using the following parameters: An energy scan range from 1100 to -10 eV; a pass energy of 160 eV; a dwell time of 100 ms and a step size of 1 eV. For the high-resolution spectra of C1s, N1s, and O1s, the scans were carried out within an energy window of 20-50 eV, a pass energy of 40 eV and a step size of 0.1 eV with a dwell time of 100 ms. The adventitious C (C1s = 284.6 eV) was used for calibration.

Supplementary Figure 10a shows the survey spectra of g-C_3N_4 and A-g-C_3N_4. Only C, N, and O were observed for both samples. Moreover, very small peaks were observed in the range of 0-200 eV, which can be assigned to the O2s signal and potential contamination (of *e.g.* Si from the glass vials used during the experiments). The O species can be ascribed to adsorbed water on the material surface. The high resolution C1s and N1s spectra are shown in Supplementary Figure 10b and 10c. The two peaks in the C1s spectra with binding energies of 284.6 and 288.0 eV can be assigned to the sp^2-hybridised C=C carbon and the sp^2 carbon bound to nitrogen in the triazine rings (N-C=N)[10], respectively. The N1s peak can be deconvoluted into three components, which can be assigned to the two-coordinated sp^2 N_{2f} in the heptriazine ring (398.4 eV), the central N atoms in the ring with the N-$(C)_3$ configuration (399.8 eV), and the three-coordinated amino functional group (400.8 eV).[10] The ratio between the N_{2f} peak area (398.5 eV) to the N_{3f} peak areas (400.8 eV) indicates the relative content of amino groups. A-g-C_3N_4 sample shows a lower ratio (5.3), representing more amino groups and less N_{2f} atoms compared to that of the g-C_3N_4 (6.8). This result agrees well with CHNS elemental analysis shown below.

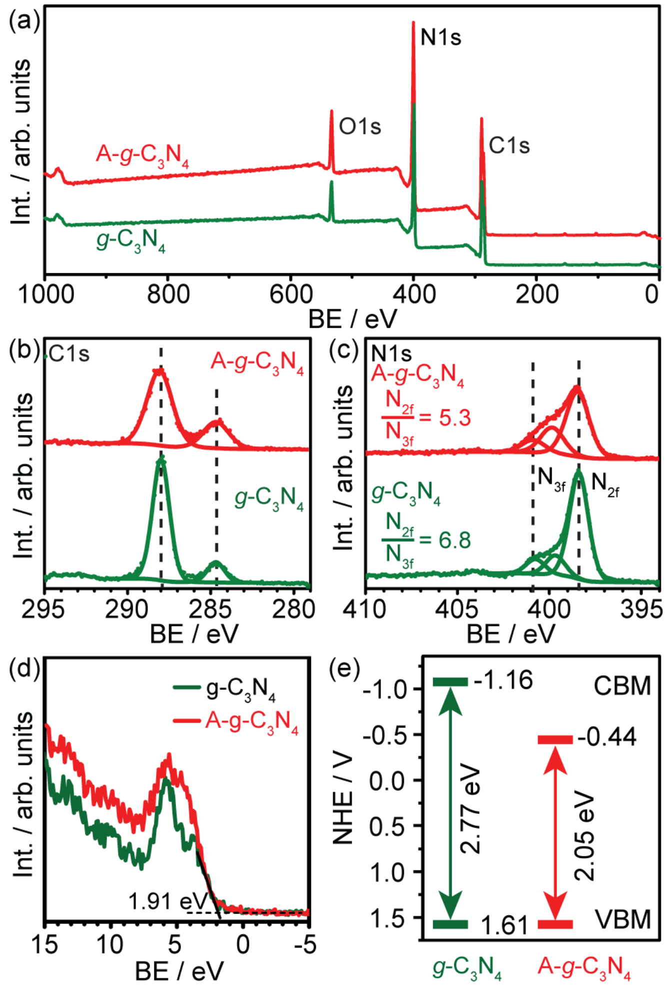

Supplementary Figure 10 XPS analysis of the photocatalyst materials. (a)-(d) XPS survey, C1s and N1s, and valence band spectra of the g-C_3N_4 and A-g-C_3N_4, respectively. (e) Estimated conduction band minimum (CBM) and valence band maximum (VBM) of both samples.

The valence band (VB) spectra indicate that the VB maximum (VBM) of both samples are located at the same position (1.91 eV, Supplementary Figure 10d). By further coupling with the DRS analysis, we have plotted the positions of the VBM and conduction band minimum (CBM) of both materials as referenced to the normal hydrogen electrode (NHE), as depicted in Supplementary Figure 10e. While the VBM of both samples are aligned to 1.61 V *vs*. NHE, the CBM of g-C_3N_4 (-1.16 V *vs*. NHE) is more negative than that of A-g-C_3N_4 (-0.44 V *vs*. NHE). Therefore the strong reduction ability of the g-C_3N_4 sample could also contribute to the enhanced photocatalytic nitrobenzene reduction performance.

The bulk chemical compositions (C, N, and H) of the g-C_3N_4 and A-g-C_3N_4 were characterised by CHNS elemental analysis using a Vario Elementar cube analyser, as shown in Supplementary Table 3. The A-g-C_3N_4 sample shows a significantly lower nitrogen content in comparison with g-C_3N_4, which is mainly due to the loss of N_{2f} atoms during the preparation process as reported in the literature[11].

Supplementary Table 3 Elemental analysis of the as-synthesised samples.

Sample	C/ wt.%	N / wt.%	H / wt.%	Stoichiometry
**g-C_3N_4**	35.2	62.0	2.1	$C_3N_{4.53}H_{1.06}$
**A-g-C_3N_4**	33.9	53.3	2.3	$C_3N_{4.04}H_{1.22}$

The detailed nanostructures of the samples were obtained using a TALOS F200A transmission electron microscope (TEM) with a TWIN lens system, an X-FEG electron source and a Ceta 16M Camera.

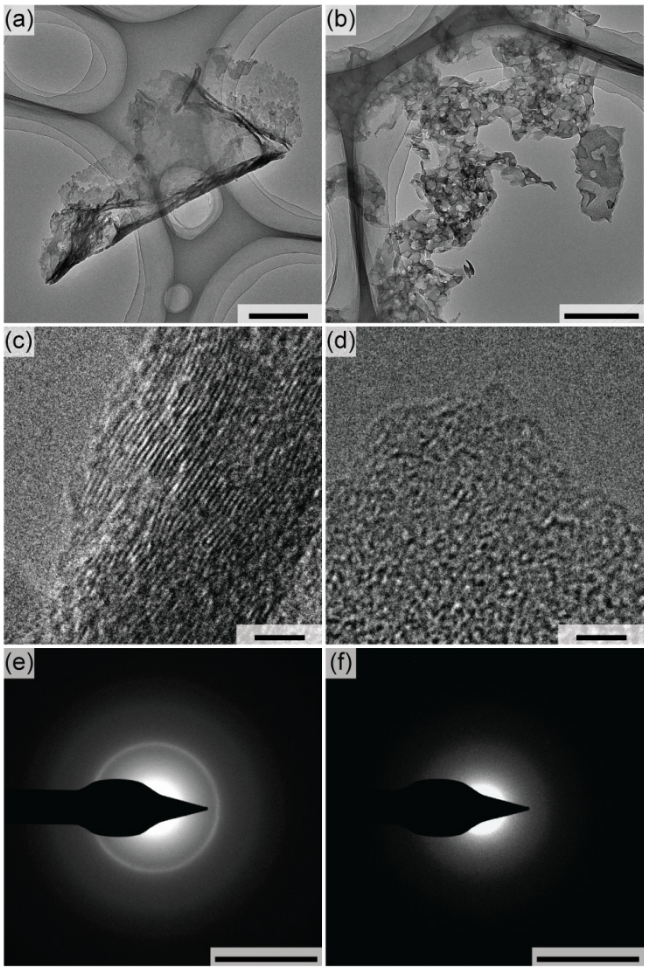

Supplementary Figure 11 Microstructure analysis of the photocatalyst. (a and b) TEM images of g-C_3N_4 and A-g-C_3N_4 (scale bar 500 nm); (c and d) HRTEM images of g-C_3N_4 and A-g-C_3N_4 (scale bar 3 nm); (e and f)SAED images of as-prepared g-C_3N_4 and A-g-C_3N_4 (scale bar 5 nm^{-1}).

As shown in Supplementary Figure 11a, the g-C_3N_4 sample has a layered morphology with some pores that were formed due to the gas release caused by the urea decomposition during the sample preparation. In contrast, a more porous nanostructure with holes larger than 50 nm is observed for the A-g-C_3N_4 sample (Supplementary Figure 11d). These big holes can be explained by the decomposition of g-C_3N_4 due to the post-heating treatment. The porous structure of the A-g-C_3N_4 sample determined by TEM is in good agreement with the BET surface area analysis as well as the structural characterisation (PXRD and XPS). In addition, the high resolution TEM (HRTEM) image (Supplementary Figure 11b) shows that g-C_3N_4 presents a partially crystalline structure, while the A-g-C_3N_4 sample (Supplementary Figure 11e) is characterised by a typical amorphous structure. The structural difference is also evidenced by the selected area electron diffraction (SAED) patterns (Supplementary Figure 11c and 11f) in agreement with PXRD.

Supplementary Discussion

We suggest that the poor performance of A-g-C_3N_4 compared to that of the g-C_3N_4 can be associated with the free adsorption energy of the photogenerated H_{ads} (ΔG_H). It was observed that the colour of the A-g-C_3N_4 reaction suspension changed from orange to brown after irradiation (Supplementary Figure 12a and 12b). The treated A-g-C_3N_4 is similar to the g-C_3N_4 treated by H_2 during hydrolysis reported previously.[12] Moreover, the suspension remained brown even after exposure to air for a long time (≥ 1 h), indicating that the hydrogenated A-g-C_3N_4 sample has a relatively high stability due to a high ΔG_H. In contrast, the colour of the g-C_3N_4 reaction suspension only changed slightly from yellow to light green, which rapidly changed back to yellow when exposed to air.

In order to verify the hypothesis that photoreduced hydrogen binds stronger to A-g-C_3N_4 than to g-C_3N_4, *post mortem* temperature programmed desorption (P-TPD) analysis of the used photocatalysts was conducted using a chemisorption analyser

(AutoChem II) coupled with a MS (OmniStar GSD, Pfeiffer). The P-TPD analysis was performed following the procedure described below:

The reaction was performed under the same conditions as the photocatalytic nitrobenzene reduction with the only difference that no nitrobenzene was added to the reaction mixture: 50 mg photocatalyst and 100 µmol KOH in 10 mL isopropanol solution was stirred in a 1 bar N_2 atmosphere at room temperature. The mixture was irradiated for 5 h with 410 nm light. When the irradiation was stopped, the suspension was kept stirring at room temperature and under vacuum conditions until it was totally dry. Finally the dry photocatalyst was loaded into a U-shaped quartz tube in a chemisorption analyser. To start the TPD test the loaded sample was heated to 500 ^{o}C at a ramp rate of 10 $^{o}C\cdot min^{-1}$ with a He flow rate of 50 $mL\cdot min^{-1}$. Meanwhile the desorbed gases were monitored by the coupled MS in real time. For comparison, the freshly prepared photocatalysts were analysed directly in the chemisorption analyser to perform the TPD test under the same conditions.

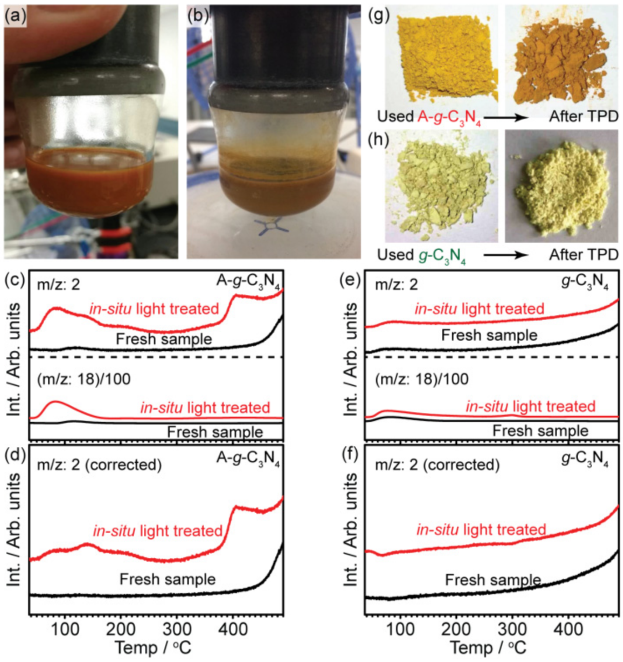

Supplementary Figure 12 P-TPD analysis of the photocatalysts. (a) and (b) Photographs of the A-g-C_3N_4–isopropanol reaction suspension before and after irradiation. (c) to (f) P-TPD of the used A-g-C_3N_4 and g-C_3N_4. (g) and (h) Photographs of the vacuum dried used A-g-C_3N_4 and g-C_3N_4 before and after P-TPD, respectively.

The raw desorption profiles of m/z= 2 and water (m/z= 18, 1% of its original intensity) of the A-g-C_3N_4 and the g-C_3N_4 samples analysed by MS are shown in Supplementary Figure 12c and 12e. It should be noted that the ionisation of water results in m/z= 18 and m/z= 2 signals with relative intensities of 100% and 1%, respectively. Therefore the TPD spectra of the desorbed H_2 should be corrected by subtracting the contribution of water (1% intensity of the m/z= 18 spectra). The profiles of the H_2 signals after subtracting the water background are shown in Supplementary Figure 12d and 12f. It can be clearly seen that the irradiated A-g-C_3N_4 showed a significant amount of surface adsorbed H_2, which desorbed in two different temperature intervals. In comparison, the fresh A-g-C_3N_4 and g-C_3N_4 with or without light treatment showed no obvious desorption of surface adsorbed H_2. The baseline corrected P-TPD data are shown in Fig. 2 in the manuscript. Furthermore, it is worth noting that after the P-TPD tests the colour of the used A-g-C_3N_4 had recovered to orange-red as shown in Supplementary Figure 12g, while no visible changes were observed for the g-C_3N_4 sample (Supplementary Figure 12g).

Based on the P-TPD studies, we conclude that the superior performance of the g-C_3N_4 originated from an optimised ΔG_H of the photogenerated H_{ads} extracted from isopropanol, as H_{ads} neither binds too strongly nor too loosely on the catalyst surface. Therefore, the H_{ads} atoms on g-C_3N_4 could be used efficiently for the following reduction of nitro-aromatic compounds. In comparison the strongly bound H_{ads} on A-g-C_3N_4 could not be transferred efficiently and be used in the reduction of nitrobenzene.

The *in-situ* XPS tests were conducted using a K-alpha setup which was coupled to a nitrogen filled glove-box ($p(O_2)$ < 0.1 ppm, $p(H_2O)$ < 10 ppm), as shown in Supplementary Figure 13a. The reaction conditions of the photocatalytic nitrobenzene reduction were identical to that of the reaction performed using a gas/vacuum line. During irradiation of 450 nm LED light, 64 µL of the suspension was collected and dropped onto the XPS sample stage at different time intervals (0, 3, 6, and 9 h). The suspension was dried in the XPS sample preparation chamber and subsequently characterised by XPS.

Supplementary Figure 13b and 13c show representative *in-situ* XPS results (0, 3, 9 h) of the g-C_3N_4 and A-g-C_3N_4, respectively. The N1s spectra distinctly showed that: after a reaction time of 3 h all the N1s peaks of the A-g-C_3N_4 catalyst were shifted to lower binding energies, which is probably indicative of a stable N-H_{ads} surface bond. No more changes were observed when the reaction continued from 3 h to 9 h; For the g-C_3N_4 sample, no obvious changes appeared during the whole reaction, which indirectly indicates the high stability of g-C_3N_4 during the reaction. Moreover, it suggests that the active H_{ads} on the g-C_3N_4 surface was consumed immediately after it was formed by isopropanol oxidation.

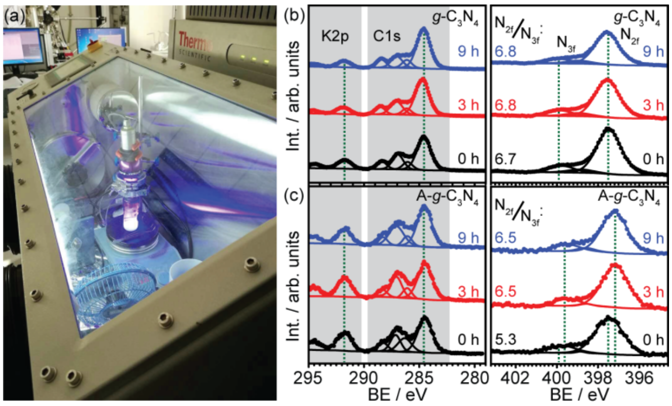

Supplementary Figure 13 In-situ XPS analysis of the photocatalyst. (a) Image of the photocatalytic nitrobenzene reduction experiments performed in the XPS coupled glove-box. (b) and (c) *In-situ* XPS C1s and N1s spectra of the g-C_3N_4 and A-g-C_3N_4. The vertical lines are a guide to the eye.

The photocurrent of both as-prepared g-C_3N_4 and A-g-C_3N_4 were analysed using a potentiostat (Autolab PGSTAT 30). A three electrode system was used for the analysis with the sample film, an Ag|AgCl, and a Pt wire as the working electrode (WE), the reference electrode (RE), and the counter electrode (CE), respectively. A 0.2 M Na_2SO_4 solution in water was used as the electrolyte. The 450 nm LED operated at 220 mW was used to excite the photocatalyst, and the photocurrent was recorded at zero external bias.

As depicted in Supplementary Figure 14, the g-C_3N_4 photocatalyst exhibits a significantly higher photocurrent compared to that of A-g-C_3N_4, indicating that the charge separation is promoted by the ordered structure of *g*-C_3N_4.

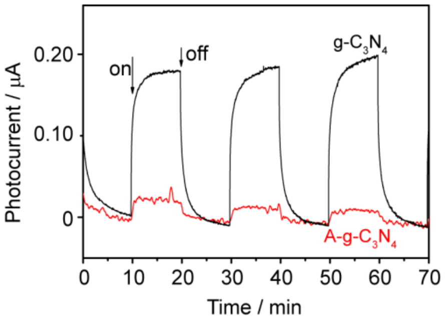

Supplementary Figure 14. Photocurrent of g-C_3N_4 (green) and A-g-C_3N_4 (red). The same mass loading was applied for both experiments.

We have also performed the reaction using aniline instead of nitrobenzene to probe the reaction mechanism. No N-N coupling products were formed, indicating that no reaction took place. This is not surprising as all reactions were performed under deaerated conditions (*i.e.* O_2 free), only allowing for reduction reaction could occur.

In order to investigate the possible reaction path of nitrobenzene reduction in our photocatalytic system, two possible intermediates (Fig. 2), *i.e.*, nitrosobenzene (NBS) and N-phenylhydroxylamine (NPH) and the product azobenzene (Azo) were purchased and used as starting reagents for photoreduction reactions under similar reaction conditions as in the nitrobenzene reduction tests. The results are shown in Supplementary Table 4. We thereby demonstrate that the g-C_3N_4 photocatalyst accelerated the coupling reaction of NBS and NPH and inhibited the further reduction of NPH to aniline. In contrast, the poor performance of P25 for the photoconversion of nitrobenzene to azo- or azoxy- benzene originates from the non-selective conversion of NBS and NPH to form aniline. Visible light irradiation does not have sufficient energy to convert azoxybenzene to aniline.

Supplementary Table 4 Photoreduction of NBS, NPH, and azobenzene using g-C_3N_4 and P25 as photocatalysts.

Photocat.	Reactant	Con. (%)	Sel. (%)	
			Azo/Azoxy	Amine
g-C_3N_4	80 μmol NBS	100	97	3
	40 μmol NBS + 40 μmol NPH	100	99	1
	80 μmol Azo	0	N/A	N/A
P25	80 μmol NBS	100	44	56
	40 μmol NBS + 40 μmol NPH	100	18	82
	80 μmol Azo	0	N/A	N/A

Reaction conditions: 50 mg photocatalyts, 80 μmol reactant and 10 mL 0.01 mol·L^{-1} KOH isopropanol solution under a 1 atm N_2 atmosphere and irradiated by LED lamps: 365 nm for 12 h for P25, 410 nm for 5 h for g-C_3N_4. The conversion and selectivity were determined by GC.

In an attempt to get further insight into possible active sites on the crystalline g-C_3N_4 surface, we have also created a model for the crystalline g-C_3N_4 system within the Molecular Orbital PACkage (MOPAC2016).[13] The PM7 Hamiltonian optimised model consists of two stripes of six linear melem units, which are bridged by N-H groups and terminated with NH_2 groups, as shown in Supplementary Figure 15. This model corresponds to a piece of the crystalline structure of melon determined by previous experimental work.[7] For systems containing only C, H and N atoms, the semi-empirical PM7 Hamiltonian has been found to have average unsigned errors of 0.14 eV (3.30 kcal·mol^{-1}) in the heat of formation, according to calculations over a set of 210 molecules.[14]

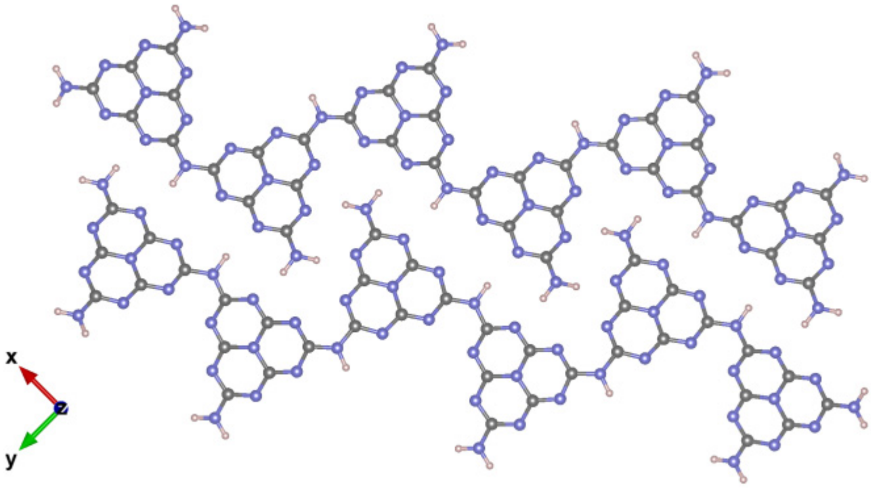

Supplementary Figure 15 Theoretical structural model for crystalline g-C_3N_4 in MOPAC2016.

Within this model system that possesses the basic adsorption sites in crystalline g-C_3N_4 (both for the sites with H-bond and edge/corner sites), we calculated H_2 adsorption energies of various adsorption sites of the two H atoms, and the energies of the most significant structures are given in Supplementary Table 5. The adsorption of two H atoms were considered together because each of the reactive steps that eliminates an oxygen atom (from the nitro group of the reactant) in the form of water requires two H atoms. Besides, two H atoms are extracted from each isopropanol molecule.

As seen from Supplementary Table 5, all the adsorption energies for the H atom pairs calculated are at least slightly less stable than free H_2. This agrees with the P-TPD results on g-C_3N_4, where no strongly bound H seems to remain after the photoreaction. The more stable adsorption energy of +0.12 eV for C3a-N2a only occurs when two H atoms sit on a corner melem unit, where the whole heptazine unit can twist to create more favourable H-bonding. Similarly, there also seems to be a preference for the H pair to adsorb on the adjacent C-N sites, with the most stable sites being the C-N bond atoms around the bridging -NH group (C3a, N2a and N3). The most stable N-N adsorption geometry (N2a and N3) is found to be ~ 0.4 eV less stable than the C-N geometries around the bridging -NH. This is because the loss of resonance energies and the disruption to the H-bonding between the melem strips can be minimised when two H atoms are bound within one melem unit and sit next to each other. In contrast, the loss of resonance energies will be greater when the two H atoms bond to N atoms in different rings.

Interestingly, when one of the H atoms is bound to the bridging –NH group, the C-N bridging bond will be sacrificed to create the N-H bond. We have not included these adsorption energies here because such bonding would destroy the 2D framework of the crystalline g-C_3N_4.

Supplementary Table 5 Adsorption energies of various H atom pairs on different adsorption sites of the melem unit

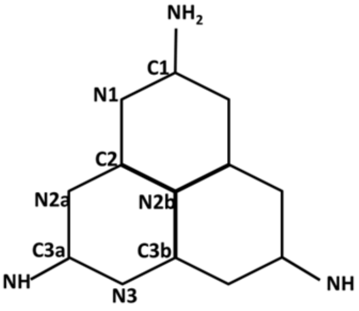

H adsorption sites	Adsorption energy (eV, relative to free H_2)
C3a-N2a	0.50, 0.12*
C3a-N3	0.51
N2a-N3	0.97
C1-N1	1.06
N2a-N2a	1.10
N3-N3	1.28
NH_2-C1	1.24
N2b-N3	2.29

*The exceptionally stable C3a-N2a geometry is only possible on the edge melem unit of the model, where the whole melem moiety can twist more easily to achieve better H bonding upon H_2 adosrption

Supplementary Note 1

We have investigated the photocatalytic synthesis of asymmetric azobenzene using two different substrates, as shown in Supplementary Table 6. Although the selectivity to the corresponding asymmetric azobenzene is not high, but rather corresponds to the statistical mixtures, we believe it might be enhanced by fine adjustment of the reaction conditions.

Supplementary Table 6 Photocatalytic synthesis of asymmetric azobenzene from two different substrates.

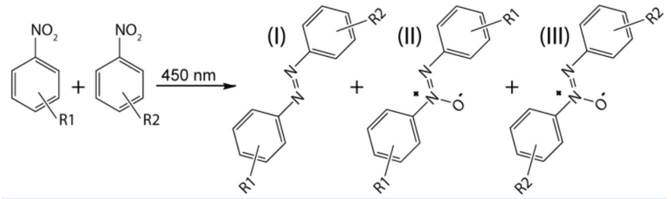

-R1	-R2	Time / h	Con. / %	Selectivity / % I	II	III
H	*p*-OCH_3	24[a]	100[c]	42	27	31
p-CH_3	*p*-OCH_3	15[b]	90[c]	51	22	27

General reaction conditions: 450 nm LED, 50 mg catalyst in 10 mL isopropanol under deaerated condition. a: 10 mW.cm^{-2}, 4 mM -R1 and 80 mM -R2, 40 mM KOH; b: 30 mW.cm^{-2}, 4 mM -R1 and 6 mM -R2, 10 mM KOH; c: conversion calculated based on the remaining amout of -R1.

Supplementary References

1. Liu, J., Zhang, T., Wang, Z., Dawson, G. & Chen, W. Simple pyrolysis of urea into graphitic carbon nitride with recyclable adsorption and photocatalytic activity. *J. Mater. Chem.* **21**, 14398-14401, (2011).

2. Kang, Y., Yang, Y., Yin, L., Kang, X., Liu, G. & Cheng, H. An Amorphous Carbon Nitride Photocatalyst with Greatly Extended Visible-Light-Responsive Range for Photocatalytic Hydrogen Generation. *Adv. Mater.* **27**, 4572-4577, (2015).

3. Su, R. *et al*. Designer Titania-Supported Au–Pd Nanoparticles for Efficient Photocatalytic Hydrogen Production. *ACS Nano* **8**, 3490-3497, (2014).

4. Su, R. TiO_2 Based Photocatalyst: From Synthesis and Characterization to Optimization and Design. Ph. D. thesis, Aarhus University, Aarhus, 2007.

5. Guo, X., Hao, C., Jin, G., Zhu, H. & Guo, X. Copper Nanoparticles on Graphene Support: An Efficient Photocatalyst for Coupling of Nitroaromatics in Visible Light. *Angew. Chem. Int. Ed.* **53**, 1973-1977, (2014).

6. Liu, J. *et al*. Metal-free efficient photocatalyst for stable visible water splitting via a two-electron pathway. *Science* **347**, 970-974, (2015).

7. Lotsch, B. V. *et al*., Unmasking Melon by a Complementary Approach Employing Electron Diffraction, Solid-State NMR Spectroscopy, and Theoretical Calculations-Structural Characterization of a Carbon Nitride Polymer. *Chem. Eur. J.* **13**, 4969-4980, (2007).

8. Kubelka, P & Munk, F. Ein beitrag zur optik der farbanstriche. *Z Tech. Phys.* **12**, 593-601, (1931).
9. Tauc, J., Grigorovici, R. & Vancu, A. Optical Properties and Electronic Structure of Amorphous Germanium. *Phy. Status Solidi* **15**, 627-637, (1966).
10. Han, Q. *et al.* Atomically Thin Mesoporous Nanomesh of Graphitic C_3N_4 for High-Efficiency Photocatalytic Hydrogen Evolution. *ACS Nano* **10**, 2745-2751, (2016).
11. Liu, P., Liu, G., & Cheng, H. Nitrogen Vacancy-Promoted Photocatalytic Activity of Graphitic Carbon Nitride. *J. Phys. Chem. C* **116**, 11013-11018, (2012).
12. Tay, Q. *et al.* Defect Engineered g-C_3N_4 for Efficient Visible Light Photocatalytic Hydrogen Production. *Chem. Mater.* **27**, 4930-4933, (2015).
13. MOPAC2016, HTTP://OpenMOPAC.net/, Version 17.186W, J.J.P. Stewart, Stewart Computational Chemistry, Colorado Springs, CO, USA.
14. PM7 Accuracy, MOPAC Manual, HTTP://OpenMOPAC.net/PM7_accuracy/PM7_accuracy.html
